# Nanomechanical Hallmarks of *Helicobacter pylori* Infection in Pediatric Patients

**DOI:** 10.3390/ijms22115624

**Published:** 2021-05-25

**Authors:** Piotr Deptuła, Łukasz Suprewicz, Tamara Daniluk, Andrzej Namiot, Sylwia Joanna Chmielewska, Urszula Daniluk, Dariusz Lebensztejn, Robert Bucki

**Affiliations:** 1 Department of Medical Microbiology and Nanobiomedical Engineering, Medical University of Bialystok, PL-15222 Bialystok, Poland; piotr.deptula@umb.edu.pl (P.D.); lukaszsuprewicz@gmail.com (Ł.S.); tamara.daniluk@umb.edu.pl (T.D.); sylwia.chmielewska@umb.edu.pl (S.J.C.); 2 Department of Human Anatomy, Medical University of Bialystok, PL-15230 Bialystok, Poland; anamiot@poczta.onet.pl; 3 Department of Pediatrics, Gastroenterology, Hepatology, Nutrition and Allergology, Medical University of Bialystok, Children’s Clinical Hospital, PL-15274 Bialystok, Poland; urszula.daniluk@umb.edu.pl (U.D.); lebensztejn@hoga.pl (D.L.)

**Keywords:** *Helicobacter pylori*, atomic force microscopy, tissue rheology, mechanomarkers, mechanobiology, histopathology

## Abstract

Background: the molecular mechanism of gastric cancer development related to *Helicobacter pylori* (*H. pylori*) infection has not been fully understood, and further studies are still needed. Information regarding nanomechanical aspects of pathophysiological events that occur during *H. pylori* infection can be crucial in the development of new prevention, treatment, and diagnostic measures against clinical consequences associated with *H. pylori* infection, including gastric ulcer, duodenal ulcer, and gastric cancer. Methods: in this study, we assessed mechanical properties of children’s healthy and *H. pylori* positive stomach tissues and the mechanical response of human gastric cells exposed to heat-treated *H. pylori* cells using atomic force microscopy (AFM NanoWizard 4 BioScience JPK Instruments Bruker). Elastic modulus (i.e., the Young’s modulus) was derived from the Hertz–Sneddon model applied to force-indentation curves. Human tissue samples were evaluated using rapid urease tests to identify *H. pylori* positive samples, and the presence of *H. pylori* cells in those samples was confirmed using immunohistopathological staining. Results and conclusion: collected data suggest that nanomechanical properties of infected tissue might be considered as markers indicated *H. pylori* presence since infected tissues are softer than uninfected ones. At the cellular level, this mechanical response is at least partially mediated by cell cytoskeleton remodeling indicating that gastric cells are able to tune their mechanical properties when subjected to the presence of *H. pylori* products. Persistent fluctuations of tissue mechanical properties in response to *H. pylori* infection might, in the long-term, promote induction of cancer development.

## 1. Introduction

Cancer disease is one of the leading causes of death worldwide, accounting for about one in six deaths. The World Health Organization (WHO) estimated that in the year 2018 cancers were diagnosed in 18 million people, and the number of cancer-related deaths exceeded 9.5 million [1]. Tumors of the large intestine and stomach are the third and fifth most common cancers, accounting for 10% and 6% of cancer cases, respectively. It should be mentioned that gastric cancer is the third most deadly cancer in the world [1]. One of the risk factors confirmed for gastric cancer is infection with the bacterium *H. pylori* [2,3,4,5,6], which is a spiral-shaped Gram-negative bacterium that infects more than half of the world’s population [2,7,8]. This bacterium colonizes the gastric mucosa and the surfaces of the gastric epithelium. It is estimated that up to 70% of the world’s population carries this bacterium, especially in developing countries [2,9,10]. *H. pylori* infection usually occurs in childhood, and when untreated, remains present in the life of the host [3,6,11]. The infection is usually associated with asymptomatic gastritis, and in the long-term it may result in more severe clinical outcomes, such as gastric ulcer, duodenal ulcer, and gastric cancer [2,3,4,12,13]. This bacterium has various virulence factors, including a cytotoxin-associated gene A (CagA), a vacuolating cytotoxin A (VacA), and various outer membrane proteins that allow this microorganism to move around and survive in the unfavorable environment of the human stomach. Moreover, CagA and VacA may deregulate the host’s intracellular signaling pathways, promoting neoplastic transformation [2,13,14]. CagA can interact with the host’s proteins to activate further signaling pathways, thereby activating the host’s inflammatory responses and cell proliferation. Activating the immune response additionally enhances the production of reactive oxygen species by increasing the oxidative stress leading to the cell and DNA damage, and thus promotes carcinogenesis. Uncontrolled cell proliferation also leads to mutations that occur during normal DNA replication of cells [2,14].

The molecular mechanism of *H. pylori*-induced gastric cancer has not been fully elucidated. It is necessary to understand all the mechanisms related to the induction and regulation of immune and inflammatory responses to *H. pylori* [2,15,16,17]. In recent years, scientific studies have appeared stating that the dysfunction of physiological processes during infection, mostly development of inflammation generates the changes in cells and tissues, which leads to changes in their rheological properties that might lead toward cancer development [18,19,20,21,22,23,24,25]. The information on the nanomechanical aspects of the pathological state of *H. pylori* gastric infection could be of key importance in the development of new bacterial eradication methods, preventive and diagnostic measures. Lately, more attention has been paid to the mechanical testing of tissue samples to understand thoroughly the physiological and pathological processes occurring at the cell and tissue levels [20,24,25,26]. All physiological processes and their dysfunctions during the development of infection are associated with structural changes that can be described at various structural/organization levels of tissues, cells, and cell organelles. These structures have specific mechanical properties. Cells or tissues frequently change their stiffness/rigidity during inflammation or the development and progression of a neoplastic disease [24,27,28,29]. These changes often precede those observed in typical histopathological examinations. The interest in the “mechanical aspects of inflammation” was possible due to the development of new techniques enabling measuring the rheological properties of biological samples—cells and tissues [20,30]. The atomic force microscopy (AFM) developed in the early 1990s offers the possibility of surface imaging at the nanoscale and nanomechanical characterization by indenting soft biological materials under physiological conditions [20,26,30]. AFM is very useful to evaluate the mechanics of biological, especially “live” or freshly isolated samples. There are also attempts to use it in the routine diagnosis of cancer [24,25,26]. The aim of the study was to assess the potential of atomic force microscopy to measure the mechanical properties of the gastric mucosa samples described as healthy and *H. pylori* infected that were collected during gastroscopy and human gastric cells culture upon exposure to heat-treated *H. pylori* cells. In addition, the study has attempted to describe possible mechanical markers of infection that could support development of new diagnostic, prevention and treatment methods of *H. pylori* infection.

## 2. Results

**Figure 1:**
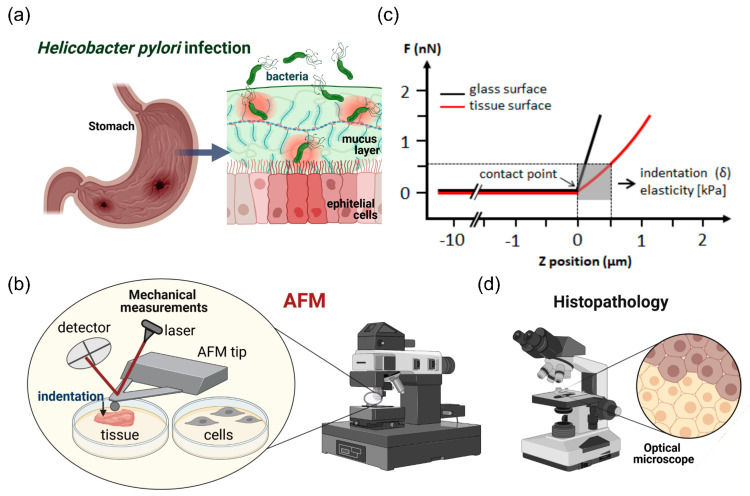
Schematic representation of the experimental setting. Mechanical properties of stomach tissues (Panel **a**) were tested using atomic force microscopy (Panel **b**) and immunohistochemical staining (Panel **d**). Panel c shows a diagram of the method to assess Young’s modulus of tissues and cells. AFM experimental setup/installation: the main AFM components are a cantilever with spherical (a bead with diameter of 4.5 μm) and pyramidal tip, a laser source, a photosensitive photodiode, and a piezoelectric scanner that can apply a compressive force to soft tissues at the nanoscale. Application of the compressive force measured as a function of a sample’s position in Z-direction induces so called force vs. distance curves (Panel **c**). The difference between the cantilever deflection on a stiff glass or hard plastic surface (blue curve) and the soft sample (red curve) describes the deformation of the tissue sample under the load, which permits determination of a sample modulus of elasticity (Young’s modulus).

In the first set of the experiments, the stiffness of healthy and infected tissues was measured using the atomic force microscope. AFM testing consisted of the series of loading-unloading cycles over the tissue’s surface with a constant force of 2 nN using a cantilever with the polystyrene bead attached (Figure 1). Details of performed experiments are included in the Materials and Methods section.

**Figure 2:**
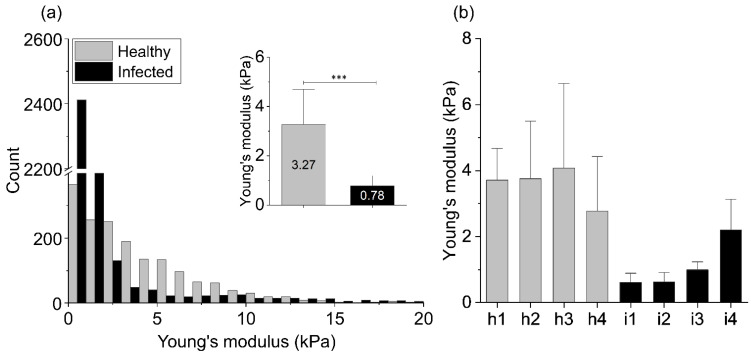
Mechanical properties of the healthy (h1–h4) and infected (i1–i4) stomach tissues: (**a**) The Young’s modulus values distributions obtained for all healthy and infected tissue samples using AFM indentation technique. The inset in Figure 2a shows the mean values of tissues’ Young’s modulus ± standard deviation to highlight the difference between healthy and infected tissues. Statistical significance was determined using two-tailed Student’s t-test for overall values. (**b**) The mean values of tissues’ Young’s modulus ± standard deviation for each healthy and infected tissue. *** indicates statistical significance at *p* ≤ 0.001.

Figure 2 shows the Young’s modulus values obtained using AFM indentation technique. Relative values of the Young’s modulus distributions from 4 healthy tissues (*H. pylori* negative, h1–h4) and 4 tissues identified as *H. pylori* positive (i1–i4), where the Young’s modulus of healthy and infected biopsies are compared. The inset shows mean values ± standard deviation for healthy and infected tissue samples. Panel b shows the mean values of tissues’ Young’s modulus for each healthy and infected tissue.

The summary histogram presented in Figure 2 shows the overall results obtained for all tested infected and healthy tissues. Distributions of the Young’s modulus values of the infected samples are shifted to lower values of the elastic modulus and a difference between healthy and infected tissue stiffness can be observed. The mean Young’s modulus value and the standard deviation calculated for healthy stomach tissue equals 3.27 ± 2.83 kPa, whereas for the infected biopsies 0.78 ± 0.82 kPa (inset in Figure 2a). Stiffness profiles observed in our study for the healthy stomach tissue are characterized by broad distribution, whereas the Young’s modulus distribution of the infected tissues is characterized by sharper peaks in the softer zones (lower Young’s modulus values). Only one of the infected tissue samples (i4) showed higher stiffness in comparison to the other infected tissues (Figure 2b; i4), but still it was softer than all healthy ones.

**Figure 3:**
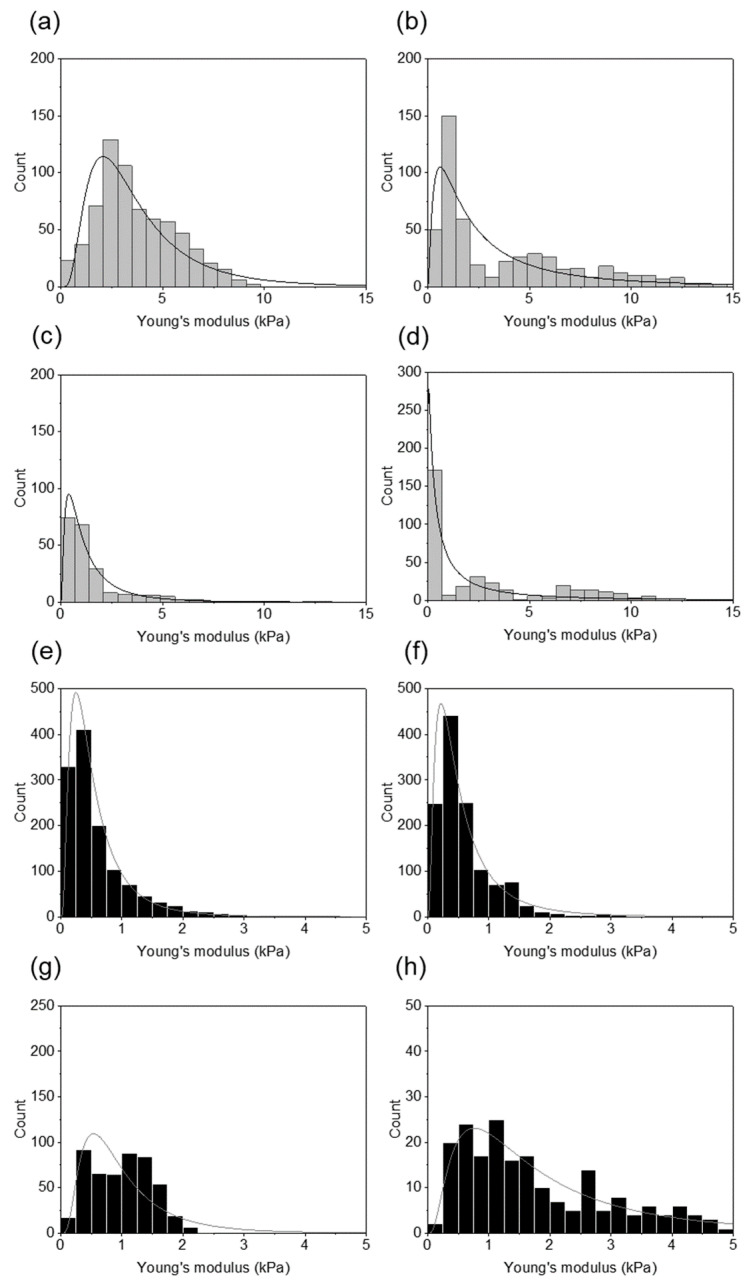
Distributions of the Young’s modulus values obtained for healthy and infected tissues using AFM indentation technique: (**a**–**d**) the Young’s modulus distributions for the healthy tissues for each patient fitted with the log normal probability density function; (**e**–**h**) the Young’s modulus distributions for the infected tissues for each patient fitted with the log normal probability density function.

Figure 3 shows distributions of the Young’s modulus values measured for each healthy and infected by *H. pylori* sample with the fitted probability density function of the log normal distribution. Young’s modulus values of the infected samples were shifted to the lower values of the elastic modulus and a significant difference between healthy and infected tissues stiffness was observed.

**Figure 4:**
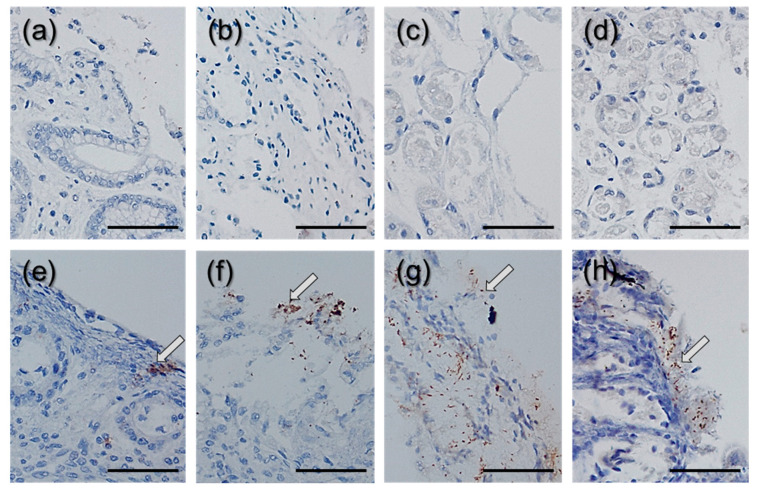
Images of *H. pylori* detected using immunostaining of the gastric bioptates: (**a**–**d**) images of stained, healthy tissues; (**e**–**h**) images of stained infected tissues. Arrows indicate the sites of bacterial colonization. A scale bar equals 100 μm.

In the next stage of the tissue evaluation, we assessed the presence of *H. pylori* within the tissue samples. Figure 4 shows microscopic pictures of biopsy samples after immunohistochemical staining with use of antibodies that recognize *H. pylori* antigens. Microscopic observations confirmed the presence of bacteria in the tissues marked as infected. *H. pylori* cells were visible in the mucosa layer and attached to the epithelial cells.

In the second stage of the study, changes in the stiffness of gastric cells exposed to *H. pylori* products were measured in time range from 0 to 72 h post *H. pylori* products addition (heat-inactivated *H. pylori* cells). AFM testing consisted of the series of loading-unloading cycles using a pyramid cantilever over the cell surfaces (near nuclei) with a constant force of 0.5 nN. 

**Figure 5:**
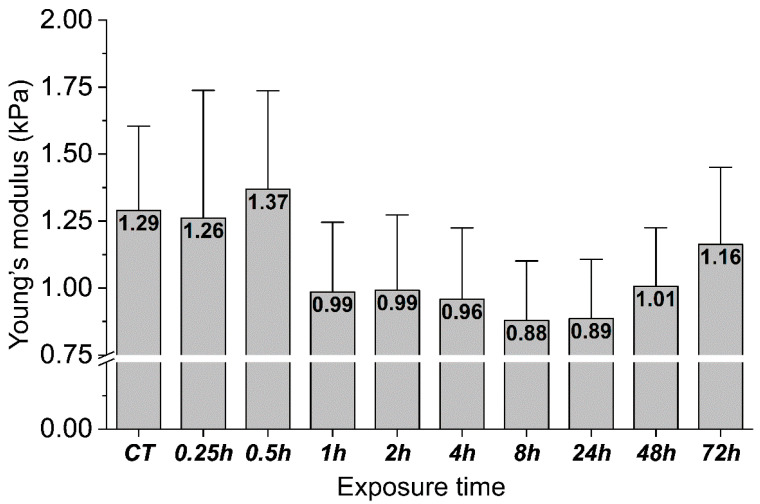
Changes in the mechanical properties of the gastric adenocarcinoma cells during exposure to heat-inactivated *H. pylori* cells. The mean values of Young’s modulus ± standard deviation (about 20 cells for one time point were measured). The inset of Young’s modulus mean values in the bars shows cells’ stiffness changes from “zero” (CT) to 72 h time point.

Figure 5 shows the mechanical response of gastric cells exposed to *H. pylori* products over time expressed as changes in their Young’s modulus. In the initial stage of infection, no apparent mechanical cell response was observed. Half an hour after the infection, slight stiffening of the cells was detected, while one hour after exposure a 23% decrease in cell stiffness was registered and lasted up to 24 h after *H. pylori* products addition. Gastric cell stiffness started to return close to its original value with time and 3 days after exposure, the cell stiffness reached 1.16 kPa.

**Figure 6:**
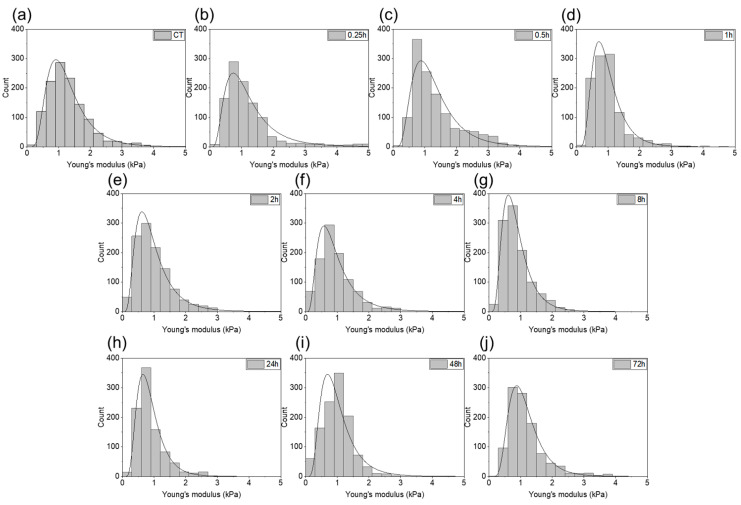
The Young’s modulus values obtained for the gastric adenocarcinoma cells during exposure to *H. pylori* products: **(a**) control sample; (**b**–**j**) post-infection samples (0.25, 0.5, 1, 2, 4, 8, 24, 48, and 72 h, respectively).

Figure 6 shows distributions of the Young’s modulus values measured for each time step of gastric cell exposed to the bacterial products with the fitted probability density function of the log normal distribution. The distribution shapes are similar. No differences in the stiffness distribution are visible, which indicates a relative cell structure and mechanical properties homogeneity.

**Figure 7:**
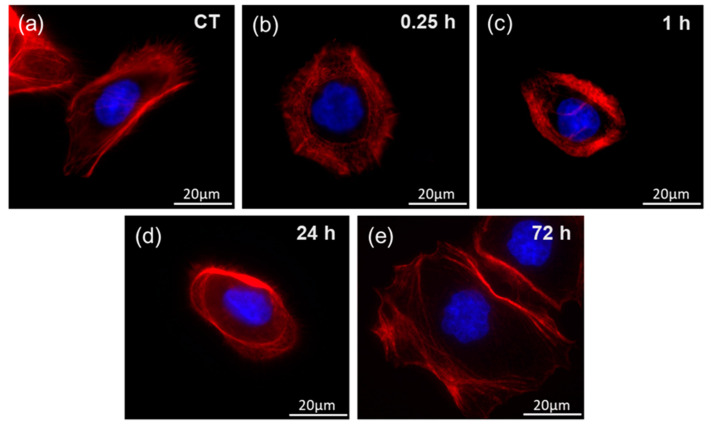
Representative fluorescent images of F-actin organization in the gastric adenocarcinoma cells during exposure to heat-treated *H. pylori* cells and corresponding Young’s modulus measurements at set time points: (**a**) control cells 0 h; (**b**) 0.25 h of incubation; (**c**) 1 h of incubation; (**d**) 24 h of incubation; (**e**) 72 h of incubation.

Figure 7 shows fluorescent images representing of F-actin organization in the gastric adenocarcinoma cells exposed to heat-treated *H. pylori* cells. Obtained Young’s modulus values correspond to those obtained for tissue samples collected from *H. pylori* infected subjects (Figure 5 and Figure 6). Changes in the internal organization of the cell’s cytoskeleton were visible within 15 min of bacterial products addition. One hour after exposure, changes in the cytoskeletons organization profoundly affected the mechanical properties of the cells (averaged value of Young’s modulus drops from 1.29 to 0.99 kPa). The cytoskeleton constituents of the cortex were changed. Actin fibers accumulate around the nucleus and the cells were less spread. The dorsal actin cortex was less uniform, a low number of stress fibers were observed. In all cases, F-actin was enriched around the cell periphery, but less in the case of cells from 15 min to 1 h after exposure. No significant changes in the structure of the cell nuclei were detected. Moreover, 72 h after exposure, the cells were spread again. Another remodeling of the internal structure of the cells was observed. Actin fibers were distant from the nucleus and the cells were more spread, a presence of stress fibers was observed. The possible mechanisms of this phenomenon are described in previous works [31,32,33,34,35], where, inter alia, effects of microbial toxins and inflammatory mediators on the F-actin organization in cytoskeleton of epithelial cells were investigated. Lipopolysaccharide (LPS) is a key component of Gram-negative bacteria cell walls. LPS has extremely strong pro-inflammatory properties. During the infection, LPS becomes the factor that triggers an inflammatory response. In order to detect pathogens the immune system is equipped with receptors called pattern recognition receptors (PRRs). These receptors are strategically localized in the cell and are key element of the immune system. The recognition of the pathogen and the initiation of the inflammatory process in epithelial cells are associated with the remodeling of the intracellular actin structure that translates into changes in the mechanical properties of the cells.

## 3. Discussion

*Helicobacter pylori* infection is one of the most common in the world and is considered an infectious disease that usually requires antibiotic treatment [1,2,4]. The knowledge about the involvement of *H. pylori* in the pathophysiology of gastrointestinal diseases is very extensive, but still incomplete. Continuous systemization of information is required to establish new treatments and prevention options via the development of new drugs and effective vaccines. Undoubtedly, understanding of the precise mechanisms of interaction between the pathogen and the host in the nanomechanical context opens up an unexplored possibility to collect new data that might serve in the development of future strategies against *H. pylori*.

The study assessed the potential of atomic force microscopy to measure the mechanical properties of the gastric mucosa in *H. pylori* infection as well as to study the response of gastric cells to infections with the use of a cell culture model. Biological structures at the organizational level, starting with cell organelles, through entire cells, tissues and ending with entire organs, have their own specific mechanical properties that influence their functions. They can be characterized by rheological parameters, such as elasticity and viscosity. Elasticity (stiffness) can be quantified using the appropriate modulus—Young’s modulus (the modulus of elasticity), which determines the resistance of a material to elastic deformation when an external stress is applied [20,36]. The modulus of elasticity of a material is defined as the slope of the stress—a strain curve in the area of elastic deformation [36]. It has been proven that the dysfunction of physiological processes during the development of diseases usually causes changes in biological structures, which translate into the changes in the mechanical properties of cells and tissues [19,20,37]. In recent years, the development of AFM enabled the nanoscale characterization of a wide spectrum of biomaterials, including human tissues [20,21,22,26,38,39]. Stiffness, along with viscosity, are the basic parameters that need to be defined.

In this study, it was observed that the values of Young’s modulus for healthy and infected gastric tissues, tested using the AFM method, are significantly different and higher for healthy tissue than for the tissue with confirmed *H. pylori* infection. Thus, it was confirmed that the dysfunction of the physiological processes during bacterial infection caused changes in the mucosa structure that translate into changes of its mechanical properties. However, earlier studies of tissues in the process of carcinogenesis indicated that pathological conditions in tissue structures affected the mechanocellular phenotype and were manifested by the changes in the tissue stiffness. Overall, neoplastic tissue with some exemption is stiffer than the normal tissue [20,21,24,25,26]. Changes in the stiffness of neoplastic tissues are closely related to the changes in the extracellular matrix, which provides structural support for the cells, enabling their proliferation, mobility and survival [40]. In the case of gastric mucosa, rheological changes may be related to deregulation of cellular functions caused by specific factors of *H. pylori* virulence. *H. pylori* is known to survive under acidic conditions due to the production of urease, which catalyzes the hydrolysis of urea to form ammonia, thus raising the pH of the environment. Rheological measurements on porcine gastric mucin (PGM) indicated that an increase in pH by *H. pylori* causes a decrease in its viscoelastic modules. Bacteria move freely at high pH, but are less mobile at low pH. It is postulated that the helicoid shape of *H. pylori* helps the bacterial cell penetrate the mucus gel, but the colonization is also influenced by the interaction of bacteria with mucins that results in changes of mucins rheological properties [41,42]. In the study [43], it was proved that the mucin characteristics, the disease state, and thus the microrheological properties, are different in different tissue locations. Additionally, this bacterium showed greater mobility in the tumor mucin solution of lower viscosity. Inflammatory processes activated by the virulence factors of *H. pylori* cells, causing gastritis and the formation of mucosa with inflammatory infiltration, might also play a role in decreasing the tissues stiffness [44,45,46]. As for the values of Young’s modules of healthy gastric tissues obtained in this study, they are consistent with the data shown previously [20,22,47]. The range of stiffness obtained for gastric mucosa is similar to data collected where an attempt was made to develop a nanomechanical profiling of other soft tissues. In all cases, the values of soft tissue stiffness measured in the conditions similar to physiological are taken into consideration. Inflammation has been identified as a risk factor for many types of cancers [48,49]. *H. pylori* infection resulting in chronic gastritis is a major factor in the initiation and development of gastric cancer [44,45,46]. *H. pylori* induces an inflammatory response both in gastric epithelial cells and in immune cells [40]. Moreover, the data suggest that *H. pylori*, via CagA, exhibits properties similar to malignant stem cells by inducing epithelial-mesenchymal transition (EMT)-like changes in gastric epithelial cells [50].

To mimic the individual gastric cell response to the presence of *H. pylori*, the gastric adenocarcinoma cell stiffness was assessed using a cell culture model. After 30 min of incubation, a slight increase in the cell stiffness was observed, followed by its decrease over the next 48 h when gastric cells were subjected to heat-treated *H. pylori* suspension. The mechanical changes were accompanied by the alterations in the internal structure of the actin cytoskeleton. Changes in the structure of cells and changes in the cell stiffness under the influence of pathogens were also observed in other studies [51,52,53]. The study [52] presents the results where human gastric neoplastic cells were treated with the tumor necrosis factor-inducing protein α (TNF-α) Tipα produced by *H. pylori*, which determines the protein as a carcinogen in the gastric epithelium. This protein influenced numerous properties, including cell migration, elongation, filopodia formation, vimentin expression, and decreased cell stiffness. Our study reports changes in the stiffness over a longer time range. Cells were simulated with heat-treated *H. pylori* for 72 h, which enabled the observation of the entire process of change in stiffness, including the return of cell stiffness close to the initial values after 48 h. This can be seen as a specific “mechanical cell relaxation”. At the molecular level, changes in the biomechanics of the gastric cells under the influence of *H. pylori* bacteria should be added to all the processes taking place in the gastric mucosa and epithelium under the influence of this and probably other pathogens, leading to inflammation and, in the long-term, to neoplasms. Viable cells are constantly exposed to mechanical stimuli from the surrounding extracellular matrix (ECM) or from neighboring cells. Mechanical stimuli are transformed into a biological response through intracellular molecular processes [54]. Cell functions and neoplastic processes are driven by the mechanical stiffness of tissues, which cells recognized through a specific molecular system [18,55]. This mechanism consists of dynamic molecular bonds between the extracellular matrix (ECM), integrins, adapter proteins, and the force-producing cytoskeleton of actomyosin. A mechanical link is created, referred to as a “molecular clutch” [55,56]. Cells use this molecular clutch to transmit and convert mechanical stimuli into biochemical signals leading to the regulation of transcription in the nucleus [55]. Mutations or abnormal activation of the sensory system of cells as well as pathological responses to mechanical stimuli are associated with myopathy, fibrosis, atherosclerosis, and cancer [54]. In this study, mechanical changes at the level of stomach cells and tissues under the influence of *H. pylori* bacteria were confirmed. These changes under the influence of chronic colonization and transfer of *H. pylori* genes in the stomach, may interfere with the transcription processes in the nuclei in the long-term and, thus, cause the risk of neoplastic formation. Cells are unable to adapt in time to continuous dynamic modifications of the microenvironment. Thus, a specific mechanopathology of the system takes place. 

The study also assessed the potential of atomic force microscopy to determine the mechanical properties of healthy and infected *H. pylori* gastric biopsies and to extend diagnostic methods. Due to the fact that it is not advisable to undertake eradication therapy without confirmation of infection, in case of possessing the tissues after biopsy, their stiffness can be tested. Unfortunately, such measurements are currently time-consuming and require high accuracy in sample preparation and testing [52]; however, efforts are being made to simplify the procedures for tissue indentation testing and to develop new methods that will allow rapid measurements of tissue stiffness [20,57,58]. Moreover, in the case of children, there are rare indications for gastroscopy and biopsy [3]. Shear rheometers can be used to measure the rheological properties of soft tissues [24,26,29], but typical gastric biopsy tissue samples are too small to perform a correct measurement. Recent studies of brain tumors using magnetic resonance elastography indicate a high potential for the dissipative feature of tissue rheology as a new marker of tissue pathology [26,59,60], especially since it is a non-invasive method. The problem in magnetic resonance elastography may be that we must measure only one tissue layer. The AFM method is sensitive enough to measure such local stiffness within a nano-areas. The AFM strategy, to assess cancer development, should include a large number of measurements at different tissue locations in order to obtain its full mechanical profile. Currently, it is not possible to base the entire diagnosis on AFM tests, but the method has great potential and can be developed to establish standard non-invasive tests. One limitation of our study that should be mentioned is the number of collected and evaluated tissue samples that were identified as *H. pylori* positive (*n* = 4). However, in studies [24,25] where AFM was used as a tool for measuring tissue stiffness, similar number of fresh tissue samples was considered adequate. The value of this study is the fact that measurements of fresh tissues were taken. From previous work [61] it might be concluded that freezing tissue samples preceding AFM measurements disrupt the internal structure, influencing their mechanical properties. Another value is the fact that biopsies from children, in which they did not occur other inflammations, including chronic ones were measured. Overall, assessment of tissue rheological parameters in the future may provide the new mechanomarkers of gastric pathology, including those related to gastric infections and cancer development; the continuation of those studies are greatly encourage.

## 4. Materials and Methods

### 4.1. Tissue Samples

In this study, the series of 20 tissues collected from children over 12 month period (2019–2020) at the Department of Pediatrics, Gastroenterology, Hepatology, Nutrition, and Allergology, Medical University of Bialystok Children’s Clinical Hospital, Bialystok, Poland, were examined. Biopsy specimens were obtained from both male and female patients at the age of 14–15 years old. Together with mechanical measurements rapid urease tests and biological tests were made. Out of the entire series of biopsies, 4 were identified as *H. pylori* positive. The results from 4 healthy tissues were selected for mechanical comparison. Collection of tissue samples was performed in accordance with an IRB protocol (R-I-002/599/2018, 31 January 2019) approved by the Bioethics Committee of the Faculty of Medicine, Medical University of Bialystok, Bialystok, Poland. All tissue samples were stored in Dulbecco’s Modified Eagle Medium (DMEM, Sigma-Aldrich, St. Louis, MO, USA) and measured within four hours post-surgery. 

### 4.2. Bacterial Strain

Clinical strain of *H. pylori* was collected from patient hospitalized in Department of Pediatrics, Gastroenterology, Hepatology, Nutrition and Allergology, Faculty of Medicine, Medical University of Bialystok, Bialystok, Poland. Bacteria were isolated from the obtained biopsies, and were cultured on BD™ Helicobacter Agar, Modified (Becton Dickinson, Franklin Lakes, NJ, USA) in microaerophile conditions (10% of CO_2_ and 5% of O_2_) at 37 °C for three days. For heat inactivation, bacteria suspended in PBS at a concentration of 10^8^ CFU/mL (O.D. ~ 0.5) were heat-treated (autoclaved at 121 °C for 30 min). 

### 4.3. Cell Culture

To determine mechanical response of cells during *H. pylori* infection, the gastric adenocarcinoma AGS (ATCC^®^ CRL-1739™, Manassas, VA, USA) cell line was used. Cells were cultured in Dulbecco’s Modified Eagle Medium (DMEM) (ATCC^®^ 30-2002™, Manassas, VA, USA) supplemented with 10% of fetal bovine serum (FBS), penicillin (50 μg/mL) and streptomycin (50 μg/mL). Cells were maintained at 37 °C in an atmosphere containing 5% CO_2_ with saturated humidity. For AFM experiments, 1 × 10^5^ cells were seeded onto 35 mm tissue culture dishes (TPP^®^, Trasadingen, Switzerland) and maintained overnight to fully adhere and spread. The following day, medium was aspirated, and cells were washed thrice with PBS. Medium was changed for serum and antibiotic free. Heat-inactivated bacteria was added to cells at final concentration of 1 × 10^6^ CFU/mL and incubated for indicated time. After incubation, cells were washed three times with PBS, medium exchanged for fresh, filtered one, and investigated with AFM.

### 4.4. AFM Measurements

In this work, mechanical properties of human stomach tissues as well as mechanical response of human gastric cells to *H. pylori* infection were tested using atomic force microscopy. Small millimeter-scale tissue samples were measured with a NanoWizard 4 Bio Science JPK Instruments Bruker atomic force microscope (AFM) (Berlin, Germany) working in the Force Spectroscopy mode. Force indentation curves were collected using a silicon nitride cantilever with a nominal spring constant of 0.62 N/m and measured spring constant in the range of 0.4–0.6 N/m using thermal tune method, with a 4.5 μm diameter polystyrene bead attached (Figure 1c). The cantilevers were manufactured by Novascan Technologies, Inc. (Boone, NC, USA). For gastric cell indentation tests the MLCT-BIO Cantilevers with a nominal spring constant of 0.07 N/m and measured spring constant in the range of 0.04–0.06 N/m, manufactured by Bruker (Camarillo, CA, USA) were used.

The bead–tissue contact area during the experiment ranged from 10 to 30 μm^2^, depending on the depth of indentation. Tissues were glued onto a Petri dish and immersed in DMEM for measurements at room temperature. To account for cantilever bending, force curves were first taken from rigid substrate, and then the stiff surface was replaced by the compliant soft tissue sample. Up to 10 indentation maps consisting of 8 × 8 points corresponding to a scan area of 10 × 10 µm were made for each sample, taken from multiple places on the tissue surface. The differences between the cantilever deflection on a control, stiff surface and the compliant biological sample describes the deformation of the tissue under the bead load (Figure 1c). By plotting the force used for sample deformation against the depth of indentation that this force induced, so-called force-versus-indentation curves were obtained. To determine the elastic modulus (i.e., the Young’s modulus) the curves were fitted to the Hertz contact model using formulas described in work [24].

In the case of cell indentation up to 22 control and conditions incubated with heat-inactivated bacteria for every time point cells were measured. Moreover, 1 × 10^4^ of the cells were seeded onto plastic TPP Petri-dishes and incubated overnight at 37 °C in an atmosphere containing 5% CO_2_. Next day, cells were washed with PBS and exposed to bacterial products for 0.25–72 h. Cell indentations for each time point were made at 37 °C in DMEM. Indentation maps consisting of 8 × 8 points corresponding to a scan area of 10 × 10 µm on the cell surface (areas near nucleus) were made. Histograms of the distributions of Young’s modulus values for each sample (tissues/cells) were prepared, and the mean values for all healthy and infected tissues and cells along with standard deviations were calculated.

### 4.5. Fluorescent Visualization of the Cells

To evaluate the organization of F-actin in the gastric adenocarcinoma cells during exposure to heat-treated *H. pylori* a fluorescent staining was performed, at condition corresponding to the Young’s modulus measurements. To do so, 1 × 10^4^ of the cells were seeded onto glass coverslips and incubated overnight at 37 °C in an atmosphere containing 5% CO_2_. The next day, cells were washed with PBS and incubated with 1 × 10^5^ CFU/mL of heat-inactivated bacteria suspended in serum and antibiotic free growth medium for 0.25–72 h. Then, cells were washed twice with PBS and fixed in 3.7% paraformaldehyde for 15 min, RT. Cells were washed again and permeabilized with 0.1% Triton X-100 for 10 min, RT. To block unspecific binding, cells were incubated for 30 min at RT with 1% bovine serum albumin (BSA) in PBS. After another wash, cells were incubated with rhodamine phalloidin solution (Thermo Fisher Scientific, Rockford, MI, USA) for 1 h, RT, in the dark. In the next step, cells were counterstained with NucBlue™ Live ReadyProbes™ Reagent Hoechst 33342 (Thermo Fisher Scientific) for 20 min, RT, protected from light. Preparation was mounted with anti-fade fluorescent mounting solution (Abcam, Cambridge, UK) and covered with glass coverslip. Cell structure visualization was performed using Leica DM4 B fluorescent microscope (Wetzlar, Germany).

### 4.6. Immunohistochemistry of the Tissue Samples

Biopsy samples were immunohistochemically treated with *H. pylori* antibodies (DAKO, FLEX Polyclonal Rabbit Anti-*H. Pylori*, Glostrup Denmark) by the streptavidin–biotin immunoperoxidase technique. The Autostainer Link 48 was used. Positive and negative controls were performed in addition to patient materials. Positive tissue control included *H. Pylori* infected gastric mucosa, the negative control was FLEX Negative Control, Mouse (Cat # IR750). Specimens were classified according to the presence of *H. pylori* under an optical microscope, (using an OLYMPUS BX53, Tokyo, Japan), and *H. pylori* positive specimens were stratified according to the respective staining pattern.

### 4.7. Statistical Analysis

The significance of differences was determined using the two-tailed Student’s t-test. Statistical analyses were performed using OriginPro 9.65 (OriginLab Corporation, Northampton, MA, USA). *p* < 0.05 was considered statistically significant. Results are the average from all force curves for all infected or healthy tissues (Figure 2a), from all force curves for each infected or healthy tissue sample (Figure 2b), or from all force curves for all measured cells come from each time point (Figure 5). Overall average values of Young’s modulus are presented as mean ± SD, where mean is the average value for the patient from all curves, and SD is a standard deviation.

## 5. Conclusions

The molecular mechanism of *H. pylori*-induced gastric cancer has not been fully elucidated and distinct studies are still ongoing. In recent years, it has been proven that the dysfunction of physiological processes during infection and the development of inflammation generates changes in cells and tissues that affect their mechanical properties. In this study, mechanical changes were evaluated in gastric biopsies and stomach cells under the influence of *H. pylori*. A comparison of the mechanical properties of the healthy and infected tissues indicates that the infected tissues are softer than the healthy ones. At the same time, the examinations of the behavior of stomach cells exposed to heat-treated *H. pylori* highlight their mechanical response. The results obtained suggest that nanomechanical parameters can be considered as the new marker of “gastric tissue mechanopathology”. The combination of standard *H. pylori* infection detection tests and mechanical measurements may place tissue rheology as a complementary procedure in the diagnosis of gastric infections. Dynamic mechanical changes at the level of gastric cells and tissues under the influence of *H. pylori* bacteria may disrupt, in the long-term, intracellular molecular processes that translate into biological responses, inducing neoplastic processes.
